# Surgery and the Surgeon

**Published:** 1916-10

**Authors:** Alfred Brown

**Affiliations:** Candler Building, Atlanta, Ga.


### SURGERY AND THE SURGEON.

**By Dr. Alfred Brown, Candler Building, Atlanta, Ga.**

Every last one amongst us appreciates the great importance attached to the earnest attempts that are constantly being exercised to enrich the profession as a whole in regard to the acute and chronic conditions that occur in the abdominal cavity.

I especially emphasize acute conditions because it is to this class of cases that the uneasiness or anxiety of the family and friends request the family physician to call a consultant. And right here is where in a great many cases all chances for the patient are exploded. It so often happens that the consulting surgeon accepts the diagnosis of the existing malady already made by the physician in charge, and that the only province over which he thinks he holds any jurisdiction is to operate, or in other words open the abdoman and look in.

This attitude of mind I do not hesitate to say is not only bad for the patient, but is the cause of so many unnecessary operations, and one of the direct causes of so much criticism being hurled upon our profession by the press.

In our daily papers we read too often: Mr. or Mrs. So and So died following an operation. Literally this is true and the public usually accepts this as it is written, never making any distinction between a preexisting cause and an amateur surgeon, thus accounting for the vials of wrath that are constantly poured upon the profession of surgery.

And, gentlemen, in spite of all this, almost every progressive physician desires surgical consultation in all cases that do not readily respond to ordinary medical or hygienic treatment. So often he hesitates to do so because from his past experience he feels that his colleague will offer no other plan than I have already referred to: make an incision and look in. It is generally believed by all that a surgeon has a better understanding of morbid processes that are taking place within the body, and that he also posesses a greater appreciation of the ultimate result of this perverted physiological action. But we also know that most of them hesitate to make any definite statement or suggest medical treatment or give any encouragement until they are permitted to explore and determine the exact condition existing therein.

Here lies the stumbling block to our progress. This is the obstacle that must be removed, or in other words this is the missing-link between the internest and the surgeon. Until the surgeon reaches a higher degree of efficiency as a diagnostition, until he is able to offer an opinion worth while or something other than the usual immediate operation, and until he has learned that the scalpel is not to be used every time some patient has a stomachache, the old family doctor will continue to believe that it is to his and his patient's welfare to seek assistance elsewhere.

Let us in as few words as possible take into consideration our foundation upon which we build our argument relative to making a diagnosis and instituting proper treatment or proper advice. You will bear me out that a careful, accurate and complete history of the patient's past and present medical, or surgical life, along with a practical understanding of its significance, rarely, if ever, will such a history fail to point out a route for further investigation. Any omission of detail will in some instances distort our clinical picture and would be very apt to throw the diagnostician off the right track.

A careful physical examination and its value is so well understood and appreciated that to enter into any detail as inspecting, palpating, and percussing, is entirely unnecessary. We all make these examinations very well and so far as the library is concerned it seems to be sufficient to come to some satisfactory conclusion. Perhaps we might here include the laboratory. This, gentlemen, while very essential, has in many cases only a limited value and in the hands of a student is worse than useless, as it will invariably lead him astray. I wish here to emphatically emphasize that a leucocytosis develops very little definite information. One urinalysis showing the presence of albumin is not sufficient to make a diagnosis of Bright's disease, nor is the presence of glucose one time an indication of diabetes any more than a diagnosis of syphilis because the patient is a negro. Much less are we justified in subjecting a patient to an abdominal operation because he shows an increase white cell count. As absurd as this sounds I have seen it done repeatedly.

A diagnosis made on physical signs alone will very often stand and when these cases respond to ordinary treatment or the proper operation performed all goes well.

The so-called therapeutic diagnosis is the big stick of the old-time doctor, and one with which he very ably defends himself and defies all comers, and the method we so often find ourselves adopting as we grow older in experience. Experience is a great teacher if we profit by it, even though our schools condemn any such practice.

What then, must be done? So far in our discussion I have mentioned very little but what can be found in any medical library and so far we probably have disposed of the majority of our cases, and in a satisfactory manner. It is to the balance of our cases that I wish to allude to, which includes all those cases where a positive diagnosis is not only difficult but almost impossible to make, and what concerns the doctor more than anything else is whether or not the patient is improving or gradually getting worse? Is an operation indicated; will it do any good if performed?

It is at this point that we need the help and support of our colleague, and it is also at this point that we need the assistance of a surgeon whose enthusiasm for using his scalpel has dwindled down to good sound scientific judgment and conservatism. If he is well informed and has had good surgical training he does and has a right to know and better appreciate the significance of the character of the pulse and its variations, the cycle of the temperature curve, the laboratory findings and its significance, and the changes that have taken place in regard to their relation to each other. Last, but very important, and I especially wish to emphasize and can only be learned by observation, is the general appearance of the patient, the expression of the face, their position and their attitude toward what has been their life interests. These points taken collectively and when properly understood, present a picture that will rarely if ever lead you astray, and when such a picture is presented or placed before the eye of a surgeon, mellow with experience and possessed of good sound surgical judgment, any doctor will not only find solace and consolation, but good genuine information, the brand he has been seeking, and in such a surgeon the medical profession and the whole populace will and must then and there only, place their confidence.

Perhaps I speak too positively, and perhaps I eulogize our seniors in excess of their merit, but any physician that does ever so little practice will agree that the number of people going about with the scars of abdominal incisions that have received absolutely no benefit and in many instances much worse off from the ordeal they were subjected to, and are condemning our profession are gradually on the increase. This condition, gentlemen, did not exist until the country became flooded with the spirit of surgical enthusiasm extending to the point of fantasy, and to-day the belief appears to be getting more general that every woman, regardless of age, suffering from any menstrual disorder should have their ovaries resected, and woe unto he who should be so unfortunate as to have an abdominal pain, regardless of what might be the cause. According to present opinion now being propogated none amongst us would care to be accused of giving such a patient any medication—we must immediately transfer them to a hospital and operate.

When it comes to a final decision it would be far better to operate a dozen times unnecessarily than to commit the grave error of overlooking one. But in the light of present times mistakes should be few, and would be fewer if we were more exacting in our deductions and placed more confidence in our ideas and observations. So often we disregard the information that we have derived from our experience to accept some wild-cat idea that is given us through various sources. Perhaps these ideas are propagated in good faith, and when confined to the community in which they are inspired may be of some value. But in this fair land of ours, and amongst a people that are still living for the joy of assisting mankind we should exercise a great deal of discretion before accepting every idea that has its source in a highly commercialized community.

